# Exploring Synergy in Combinations of Tumor-Derived Vaccines That Harbor 4-1BBL, OX40L, and GM-CSF

**DOI:** 10.3389/fimmu.2017.01150

**Published:** 2017-09-19

**Authors:** Andrea J. Manrique-Rincón, Camila M. Beraldo, Jessica M. Toscaro, Marcio C. Bajgelman

**Affiliations:** ^1^Brazilian Biosciences National Laboratory (LNBio), Brazilian Center for Research in Energy and Materials (CNPEM), Campinas, Brazil; ^2^Medical School, University of Campinas (UNICAMP), Campinas, Brazil; ^3^Institute of Biology, University of Campinas (UNICAMP), Campinas, Brazil

**Keywords:** immunotherapy, 4-1BB, OX40, GM-CSF, tumor-derived vaccines, cancer immunotherapy

## Abstract

Recent studies have demonstrated that combination of modulatory immune strategies may potentiate tumor cell elimination. Most strategies rely on the use of monoclonal antibodies that can block cell surface receptors to overcome tumor-induced immunosuppression or acting as costimulatory ligands to boost activation of T cells. In this study, we evaluate the use of combinations of genetically modified tumor-derived cell lines that harbor the costimulatory T cell ligands 4-1BB ligand, OX40L, and the cytokine GM-CSF. The aim of these treatments is to boost the activation of T cells and the elimination of cancer cells. These tumor-derived cells are able to activate or reinforce T cell activation, thereby generating a potent and specific antitumor response. We developed a high-content *in vitro* imaging assay that allowed us to investigate synergies between different tumor-derived cells expressing modulatory immune molecules, as well as the influence on effector T cells to achieve tumor cell death. These results were then compared to the results of *in vivo* experiments in which we challenged immunocompetent animals using the B16F10 syngeneic model of melanoma in C57BL6 mice. Our results suggest that there is a substantial therapeutic benefit to using combinations of syngeneic tumor vaccines that express immune modulators. In addition, we observed that combinations of tumor-derived cells that expressed costimulatory ligands and GM-CSF induced a long-term protective effect by preventing cancer development in both cured and rechallenged animals.

## Introduction

The development of strategies that induce T cell immune modulation has significantly enhanced the ability to treat cancer. The use of antibodies to block cell surface receptors known to be associated with lymphocyte immune suppression, such as CTLA4 and PD-1, has demonstrated a high degree of efficiency and clinical applicability. Although the outcomes of studies involving these therapies are encouraging, numerous cases of treatment resistance have been documented, leading therefore, to a need for new therapeutic approaches (1–3).

In addition to blocking antibodies, which are used as checkpoint inhibitors, antitumor responses can also be enhanced by agonists that signal costimulatory receptors, such as TNFRSF receptors promoting cell proliferation, inflammatory activity, and cytotoxicity. Among this class of costimulatory receptors, 4-1BB (also known as CD137 or TNFRSF9) promotes survival, clonal expansion, and the enhancement of activated T cells (4, 5). A substantial increase in the number of memory cells was observed in transgenic animals that expressed the 4-1BB ligand (4-1BBL) and in animals that were stimulated using an anti-4-1BB agonist antibody (6). The 4-1BB receptor is also expressed in dendritic cells and its stimulation leads to increased levels of the cytokines IL6 and IL12, as well as the ligands B7-1 and B7-2A which can bind to CD28 to costimulate T cells (7). A 4-1BB agonist antibody has been shown to stimulate the immune system to eliminate established tumors in animal models (8–10). In light of these results, a human Phase I trial was performed in which human anti-4-1BB monoclonal antibodies were administered to patients with encouraging results, but a subsequent Phase II clinical trial reported severe adverse reactions that resulted from antibody toxicity, leading to the withdrawal of the protocol (11, 12). The recent data suggest that reducing the dose of this antibody and combining it with other therapies may improve its clinical benefits (13). Human clinical trials also have shown that melanoma-derived cells engineered to express 41BB-L boosted the CD8-mediated antitumor response (14).

OX40 is another costimulatory receptor that also belongs to the TNFRSF family. OX40 (also known as CD134 or TNFRSF4) is constitutively expressed on the surface of activated T cells. Costimulation of OX40 induces proliferation and boosts lymphocyte activation. OX40 signaling enhances T cell longevity. A high level of OX40 expression in tumor-infiltrating lymphocytes has been correlated with decreased metastasis and better prognoses in patients (15, 16). Studies have also demonstrated that using an agonist antibody that targets the OX40 receptor may inhibit the FoxP3 transcription factor, which is associated with the maintenance of an immune suppressive phenotype in regulatory T cells (Tregs) (17, 18). Data in the literature indicate that anti-OX40 antibodies may cause Treg depletion (19, 20). The Phase I clinical trials have demonstrated that treatment with an anti-OX40 agonist antibody increased lymphocyte antitumor activity (21, 22).

Therapeutic strategies aimed at costimulatory T cells and increasing antigen presentation can act in synergy. In this way, the activation of dendritic cells *via* the cytokine GM-CSF can induce, for example, CTL activation. Genetically modified syngeneic tumor cells that express the cytokine GM-CSF have been used as an anti-tumor vaccine that inhibit the formation of tumors in animals (23). This effect in animals was surprising, but the same result was not observed in clinical trials, indicating that it is necessary to improve therapeutic strategies (24).

Since T cells play a major role in eliminating cancer cells, *in vitro* assays can be used to evaluate enhancement of T cell activity, thereby investigating therapeutic benefits of new approaches. A gold standard assay for measuring the activity of CTLs is based on quantification of the chromium (51Cr), released by tumor cells as they die by the action of the CTLs (25). An alternative to this assay consists in quantifying the incorporation of tritiated thymidine ([3H]-TdR) into target cells (26). To overcome handling issues associated with the use of radioactive materials, other methodologies have been developed that employ measurement of leaking enzymes of dead cells, such as lactate dehydrogenase, associated with enhancement of toxicity (27) and accurate methodologies like the ELISPOT, which allows profiling of T cell response and quantification of cytokines (28). There are also flow cytometry-based methods which use 7-AAD DNA-labeled target cells (29) or even bioluminescence assays based on luciferase-expressing target cells, which are robust and faster than a Chromium assay (30). The fluorolysometric based assay can employ GFP-expressing cells as target cells. In this way, the killing ability of effector T cells can be estimated by flow cytometry, quantifying GFP-positive cells, with a fluorescence microscope to count GFP-positive cells, or even by a fluorescence plate reader measuring the leak of GFP from dead cells. The fluorolysometric assay is highly sensitive when compared to other assays that use radioactive materials or substrates for bioluminescence reactions (31).

In this work we developed a high-content imaging *in vitro* assay that allows exploration of the cytotoxicity mediated by T cells, induced by immunomodulatory antitumor vaccines. This assay is based on genetically modified tumor cells that simultaneously coexpress a single immunomodulator and the GFP reporter gene. The immunomodulatory GFP-expressing cells can be combined and cocultivated with T cells. If T cells are costimulated, killing of the immunomodulatory target cell is enhanced. The GFP marker is used as a parameter to count live cells by the high-content imaging system.

This *in vitro* assay provides three possibilities to explore: (i) monitoring mediated CTL killing of target cells, (ii) assessment of CTL profiling by flow cytometry, and (iii) quantification of cytokines in the supernatant. In this manner, the high-content imaging assay allowed exploration of the synergistic combination of tumor-derived cells that harbor immunomodulators with the aim of enhancing antitumor responses. We also performed assays using C57BL6 immunocompetent animals that were challenged with syngeneic melanoma-derived B16 tumors. These combinations of tumor-derived vaccines may provide a substantial therapeutic benefit, contributing to the development of new approaches to treating human cancer.

## Materials and Methods

### Retroviral Vector Preparations

The cDNA encoding the immunomodulators OX40L and 4-1BBL was amplified by PCR from splenocytes isolated from C57BL6 animals and cloned into pCL retroviral vectors (32). The cDNA of eGFP was isolated from FUGW lentiviral vector (33) and cloned into pBabe retroviral vector (34). Virus preparations were generated by transient transfection on 293 T cells and titrated by flow cytometry (35) by Viral Vector Laboratory at LNBio—CNPEM.

### Establishment and Culture of Cell Lines

All the cell lines were derived from the poorly immunogenic mouse melanoma cell line B16F10. Cell cultures were transduced using a retrovirus and selected with G418 or puromycin. The pCL vectors encode G418 resistance and the pBabe vector encodes puromycin resistance. The G418-resistant clones were analyzed by flow cytometry using antibodies anti-OX40L (eBIOSCIENCES clone RM134L) and anti-4-1BBL (eBIOSCIENCES clone TKS-1). We have chosen high-expression clones to establish the cell lines B16-0X40L and B16-41BBL. Next, we transduced B16-4-1BBL, B16-OX40L, and B16-GM-CSF (kindly provided by Dr. Glen Dranoff, Harvard, USA) using the retroviral vector pBabe-eGFP, which also harbors a puromycin selection cassette. Clones were analyzed using flow cytometry to select cells with a high level of GFP expression and also a high expression level of immunomodulators like 4-1BBL and OX40L. The B16-GM-CSF was also analyzed by flow cytometry for GFP and GM-CSF using a quantitative assay to determine secreted GM-CSF by ELISA (duo set ELISA kit, R&D).

### Mice

C57BL/6 mice (female, 8 weeks old, average weight 20 g) were purchased from CEMIB-UNICAMP, maintained in microisolator cages and treated in accordance with CNPEM Laboratory Animal Care regulations. The experiments completed during this study were approved by the Animal Care and Use Committee of the CNPEM, protocol CEUA—15/2015.

### Primary T Cell Isolation

In brief, primary CD4 and CD8 T cells were isolated from splenocytes using negative selection *via* the Easysep mouse CD4+ or CD8+ T cell enrichment kit (STEMCELL Technologies). The purity of CD4 and CD8 was tested after isolation by flow cytometry and was higher than 90%. These CD4 and CD8 T cells were activated for 24 h with CD3e (TONBO biosciences clone 145-2C11) and CD28 (TONBO biosciences clone 37.51) at a concentration of 1 μg/mL. Cells were cultivated in a complete medium (CM) containing RPMI (1% penicillin/streptomycin, 1% HEPES, 1% sodium pyruvate, 1% non-essential amino acids, 1% glutamine, 10% bovine fetal serum, and 50 µM β-mercaptoethanol).

### *In Vitro* Assays

Cells were cultivated into 96-well plates. Plates were seeded with a total of 1,400 adherent B16-derived cells that harbored GFP and immunomodulators, as indicated. After the cells were incubated for 24 h, 1,400 freshly isolated splenocytes, CD4, or CD8 cells were added, as indicated.

The cocultured cells were incubated with CO_2_ at 37°C for the indicated times. The CM was removed from the plates and the cells were then fixed in 4% paraformaldehyde and stained with DAPI (Sigma). The plates were scanned (27 fields-of-view/well) using an Operetta HTS imaging system (PerkinElmer) equipped with a 20× air objective lens. The excitation channels 360–400 and 460–490 and the emission filter channels 410–480 were used for eGFP, while 500–550 was used for DAPI. The images were then analyzed using Columbus (version 2.4.0 PerkinElmer).

### Flow Cytometry

Isolated or cultured cells were harvested, centrifuged at 300 *g*, and resuspended in 5% FBS-1× PBS. They were then stained with the indicated antibodies. The tumor-infiltrating lymphocytes were isolated from mice tumors, which were removed and mechanically dissociated by pushing tissue through a 70 µm strainer. The cells were then treated with ACK for 5 min on ice, washed with 1× PBS and centrifuged. We subsequently separated a fraction enriched with TILs using a 100% Ficoll Isopaque gradient. Then the cells were washed, resuspended in 5% FBS-1× PBS and stained with CD4 and CD8. After fixation and permeabilization of cells using Transcription Factor Staining Buffer Kit (Tonbo) we used an antibody against FOXP3. We used the following antibodies conjugated to fluorophores: CD4-FITC (TONBO biosciences clone GK 1.5), CD8a-PE (Biolegend clone 53-6.7), FOXP3-APC (eBIOSCIENCES clone FJK-16S) and isotype IgG2a, kappa-APC.

### Tumor Inoculation and Treatment

Mice were subcutaneously (sc) injected in the right flank with 5 × 10^4^ B16F10 cells in 100 µL of PBS. They were injected sc into the left flank on days 1, 4, and 7 with 1 × 10^6^ irradiated (50 Gy) B16F10 cells that expressed GM-CSF (Gvax), OX40L, 4-1BBL, or a combination of these cell lines (*n* = 10). We injected the total amount of 1 × 10^6^ irradiated immunomodulatory cells into each mouse, per vaccination, employing 5 × 10^5^ of each cell line when combining two cell lines and 3.33 × 10^5^ of each with the triple combination of GM-CSF + OX40L + 4-1BBL.

Tumor growth is expressed as the percentage of tumor-free mice among all injected mice on all days. Tumor size was measured using a caliper and calculated using the following equation: [(longest diameter × shortest diameter × diagonal diameter × 3.141599)/6] in cubic millimeters. Mice in which no tumors were detectable were rechallenged sc with 5 × 10^4^ B16F10 cells in the lower left flank and monitored for tumor growth.

### Histology and Immunofluorescence

Dissected B16F10 mouse tumors were embedded in Tissue-Tek OCT and frozen in liquid nitrogen. Sectioned specimens were washed with 1× PBS (pH 6.8) and incubated with glycine (0.1 M) for 30 min followed by incubation with 1% BSA for 1 h. These samples were then incubated overnight with anti-rat CD8a (eBIOSCIENCES) with a 1:100 dilution at 4°C. The slides were subsequently washed and incubated with secondary anti-rat conjugated to FITC antibodies (Invitrogen) with a 1:500 dilution at room temperature for 1 h. Nuclear staining was performed using Hoechst 33258 (Sigma-Aldrich) according to the manufacturer’s instructions. Images were captured using a Leica Confocal Microscope and images were analyzed using LAS AF software (LEICA).

### Statistical Analysis

All data were analyzed using Prism 7.0 (GraphPad software). Statistical significance was determined using one-way ANOVA followed by Dunnett’s multiple comparisons test. Tumor survival data were analyzed using the Kaplan–Meier method. The log-rank Mantel–Cox test was used to compare survival curves between different groups. Graphs show the mean and error bars, indicating the SEM of two to four independent experiments performed on different days.

## Results

### Combinations of Tumor-Derived Cells Expressing 4-1BBL, OX40L, and GM-CSF Act Synergistically Enhancing the Activity of CTLs

We developed a high-content *in vitro* imaging assay that allowed us to evaluate the efficiency of antitumor vaccines and combination of costimulatory molecules. Tumor cells that expressed GFP and immunomodulators were incubated with CD4, CD8, or splenocytes. If tumor cells were killed, it suggested a therapeutic benefit. To estimate the therapeutic benefit of monotherapies as tumor-derived vaccines, the tumor cells were incubated with activated T cells or non-primed splenocytes for 24, 48, 72, and 96 h and then analyzed using high-content imaging to count the remaining live cells (Figure S1 in Supplementary Material).

**Figure 1:**
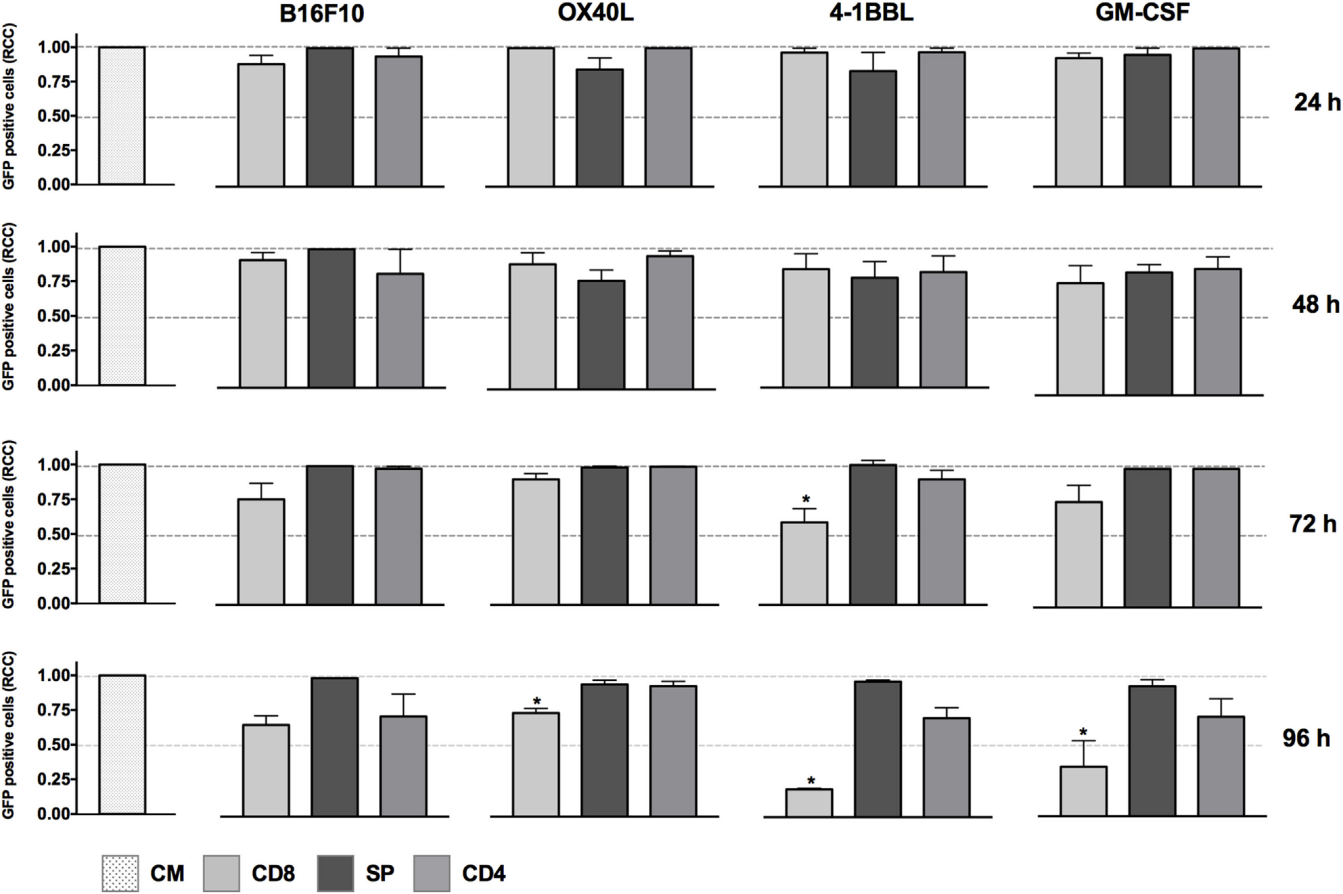
*In vitro* assay to evaluate cytotoxicity induced by cell lines harboring single immunomodulators. Graphs represent GFP-positive cells that were counted using a high-content imaging system. We tested tumor-derived cell lines that harbored single immunomodulators. The immunomodulators are indicated above the graphs. Immunomodulatory B16-GFP cells were cocultivated as shown below each graph. CM, complete medium; CD4, primed CD4 T cells; CD8, primed CD8 T cells; SP, non-primed splenocytes; RCCs: relative cell counts normalized to the counts in the CM control. Graphs of mean and SEM. ANOVA and Dunnett’s multiple comparisons against CM (*P ≤ 0.005). Results from three independent experiments performed in triplicates.

The cytotoxic activity of CD8 T cells was boosted in the presence of tumor-derived cells expressing 4-1BBL when employed as single vaccines, as shown in Figure 1. In contrast, we did not see any effect with the non-primed splenocytes using the single vaccines and just a slight reduction of tumor cells mediated by CD4 T cells in the conditions of GM-CSF and 4-1BBL at 96 h.

**Figure 2:**
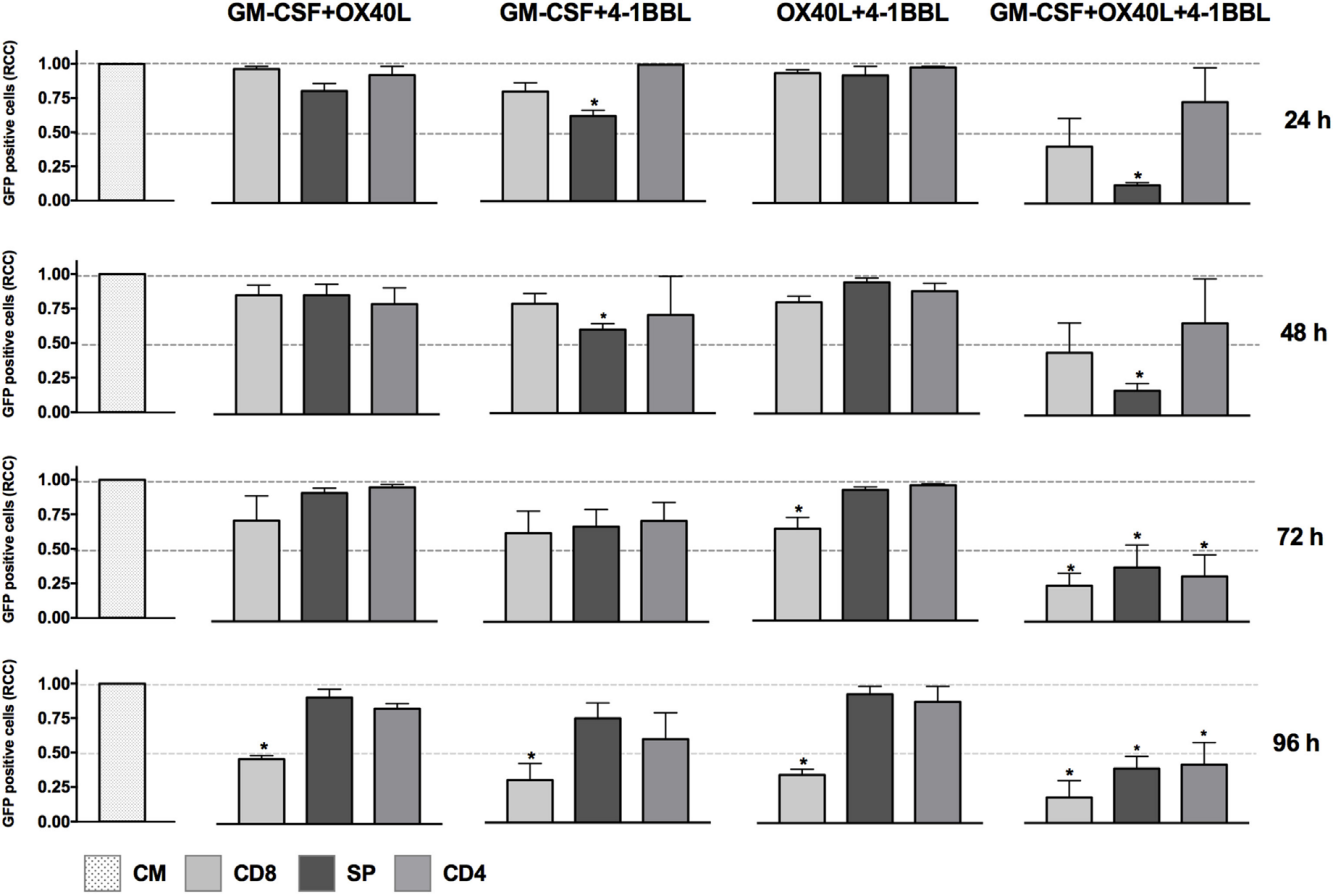
*In vitro* assay to evaluate cytotoxicity induced by the combination of cell lines harboring combination of immunomodulators. Graphs represent GFP-positive cells that were counted using a high-content imaging system. We tested combinations of cell lines that harbored immunomodulators. The immunomodulators are indicated above the graphs. Immunomodulatory B16-GFP cells were cocultivated as indicated below each graph. CM, complete medium; CD4, primed CD4 T cells; CD8, primed CD8 T cells; SP, non-primed splenocytes. RCCs: relative cell counts normalized to the counts in the CM control. Graphs of mean and SEM. ANOVA and Dunnett’s multiple comparisons against CM (*P ≤ 0.005). F10: parental B16F10 cells. RCCs: relative cell counts normalized to the counts in the CM. Results from three independent experiments performed in triplicates.

**Figure 3:**
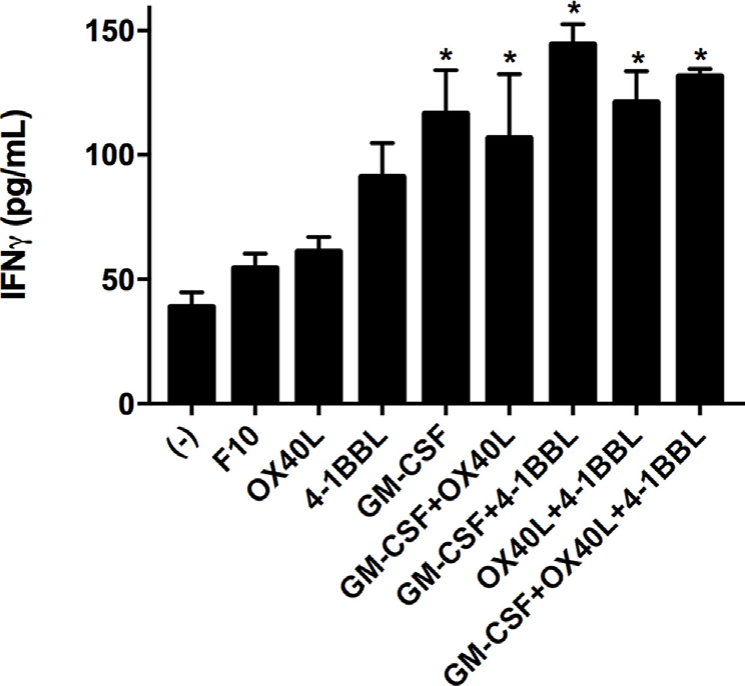
Tumor vaccines induce IFN gamma production by CD4 T cells. CD4 T cells were cocultivated with the indicated tumor-derived cells harboring immunomodulators. Concentrations of interferon gamma were determined by Elisa after 96 h of incubation. (−) Only CD4 T cells. Mean of three measurements; error bars indicate SEM, ANOVA, and Dunnett’s multiple comparisons against F10 (*P < 0.05). Experiment performed in triplicate.

We next tested all the possible combinations of B16F10-derived cells expressing GM-CSF, 4-1BBL, and OX40L (Figure 2). We observed that combination of two tumor-derived cells, harboring immunomodulators, induced a significant CD8 T cell mediated cytotoxicity. However, the response of CD4, CD8, and splenocytes was improved when they were in contact with the triple combination of tumor-derived cells, showing an early and strong induction of cytotoxicity. Strikingly, we observed a cytotoxic effect mediated by immunomodulation of CD4 T cells after 72 h. To better understand this observation of cytotoxicity, we performed an INF-γ test with supernatants of preactivated CD4 T cells that were incubated with target cells, finding the highest expression for the double combination GM-CSF + 4-1BBL followed by the triple combination of GM-CSF + OX40L + 41BBL (Figure 3).

### The Combination of Immunomodulatory Tumor-Derived Cells Boosts T Cell Activation and Enhances Antitumor Response

**Figure 4:**
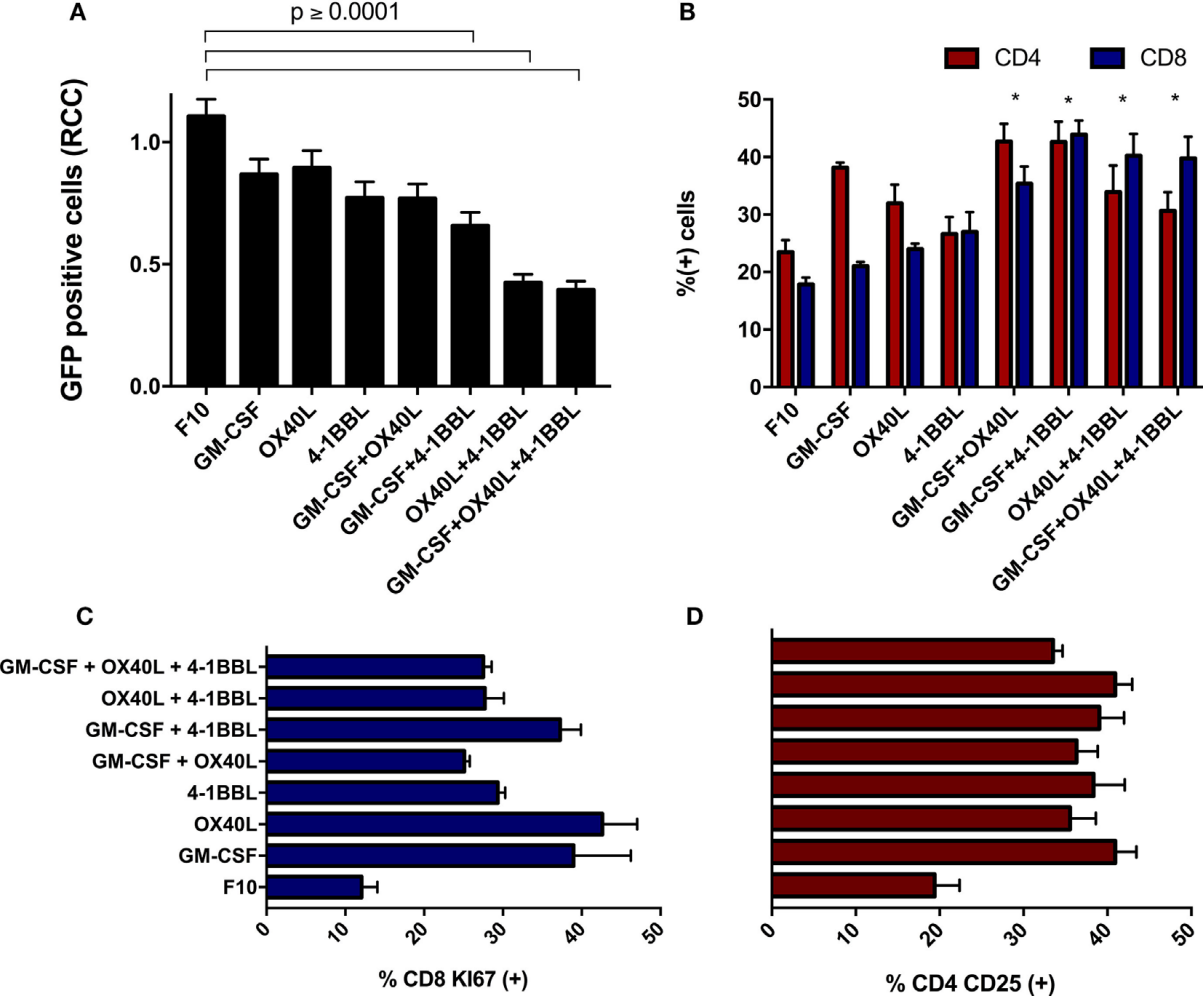
Immunomodulatory B16-derived cells boost T cell cytotoxic activity *in vitro*. **(A)** Freshly isolated splenocytes were cultivated with the indicated tumor-derived cells harboring immunomodulators, the graph indicates remaining live tumor cells after incubation with these splenocytes. **(B)** Flow cytometry of primed splenocytes showed an enrichment of CD8 and CD4 T cells for all immunomodulatory combinations (two-way ANOVA *P < 0.0001). **(C)** Freshly isolated CD8 T cells were incubated with B16F10 immunomodulatory cells, following Ki67 staining. **(D)** Freshly isolated CD4 T cells were incubated with B16F10 immunomodulatory cells, following CD25 staining (all comparison of CD4-CD25 and CD8 against F10 made with one way ANOVA and Dunnett’s multiple comparisons had a *P* value ≤0.005). Graphs of mean and SEM. One way ANOVA and Dunnett’s multiple comparison tests against F10. F10: parental B16F10 cells. RCCs: relative cell counts normalized to the counts in the complete medium (CM). Results from three independent experiments performed in triplicates.

When freshly isolated splenocytes were first incubated with immunomodulatory B16F10-derived cells and then added to parental B16F10-GFP cells that lacked immunomodulators, we observed a substantial cytotoxic effect on tumor cells. We detected a stronger cytotoxic activity using the combinations of cells harboring 4-1BBL + OX40L, GM-CSF + OX40L, and 4-1BBL (Figure 4A). An enrichment of CD4 and CD8 T cells was observed in all the combinations (Figure 4B), suggesting a boost in the proliferation of these subsets. We also observed an overall increase in the proliferation of CD8 T cells (Figure 4C) and an increased activation of CD4 T cells (Figure 4D) in all the cells expressing immunomodulatory molecules.

### The Combination of Immunomodulatory Tumor-Derived Vaccines Enhances Tumor Rejection *In Vivo*

**Figure 5:**
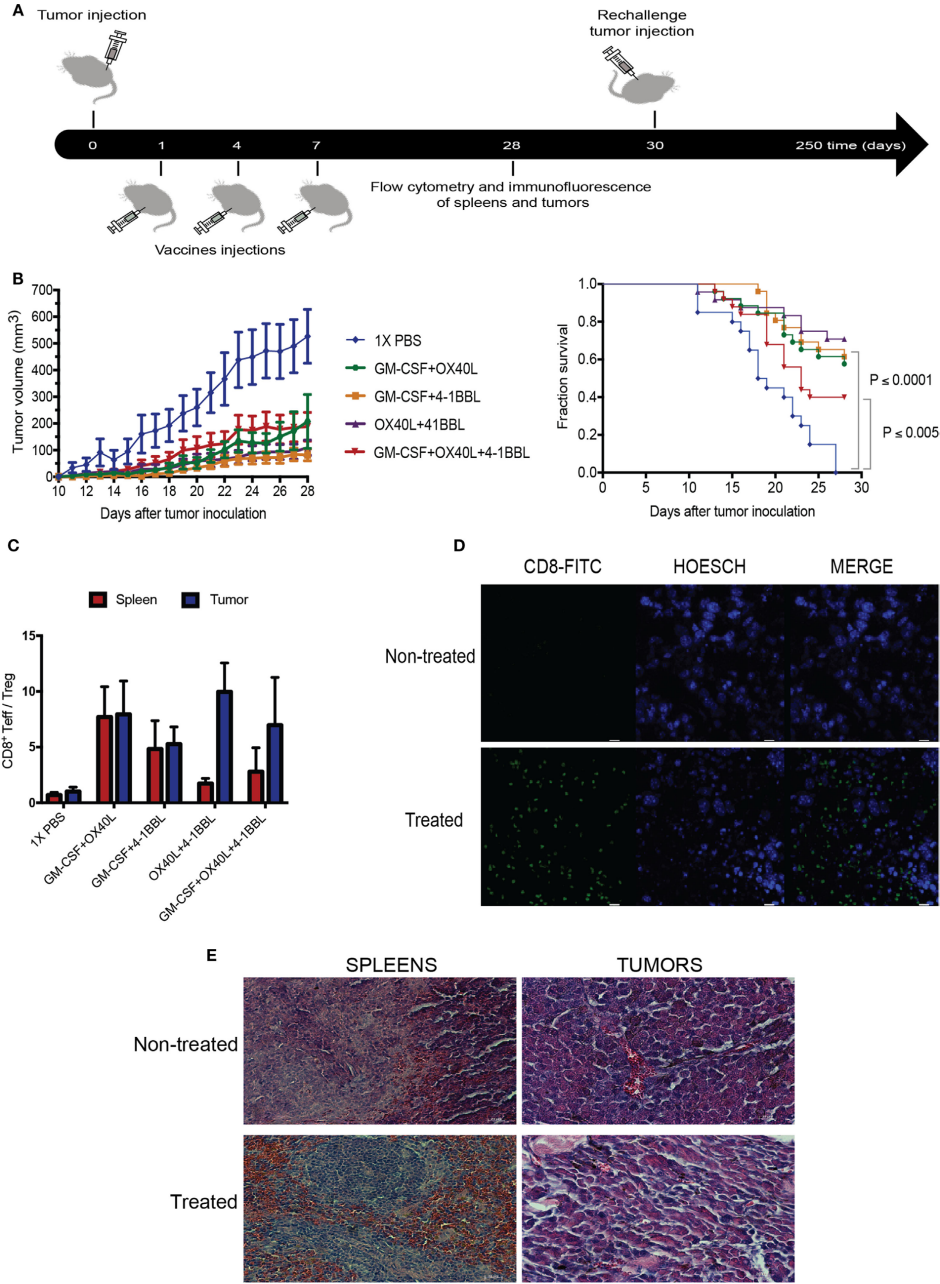
Combination of tumor-derived vaccines harboring immunomodulators enhance antitumor response. **(A)** Experimental design for *in vivo* experiment, Tumors were injected on day 0, following injections of irradiated immunomodulatory B16-derived cells on days 1, 4, and 7. On day 28, animals were sacrificed, performing analysis of lymphocytes in tumors and spleens by flow cytometry and immunofluorescence. Tumor-free mice were rechallenged on day 30 and monitored for more than 250 days. **(B)** Tumor growth and survival curves in C57BL/6 mice bearing subcutaneous B16F10 tumors, mice were treated with PBS or 1 × 10^6^ cells of different combinations of irradiated immunomodulatory B16-derived cells. These cumulative survival curves and tumor progression graph represent three independent experiments (*n* = 10 mice per group) the log-rank Mantel–Cox test was used to compare survival curves between different groups. **(C)** An analysis of tumor infiltrate lymphocytes and lymphocytes in spleens using flow cytometry reveals that the ratio of CD8/Tregs was increased by all immunomodulatory combinations. **(D)** Immunofluorescence of tumors reveals CD8 enrichment in animals treated with immunomodulatory vaccines. (This image is representative of all conditions challenged with double and triple combinations since no significant differences were observed among these groups.) **(E)** Histological analysis with hematoxylin–eosin of tumor and spleens of animals treated with the immunomodulatory vaccines. (This image is representative of all conditions challenged with double and triple combinations since no significant differences were observed among these groups.) and non-treated mice. Data are shown as the mean ± SEM. The ratio of CD8/Tregs equal to CD8 positive cells divided by FOXP3 positive cells (CD8^+Teff^/Treg ratio). Results are pooled from three independent experiments (*n* = 10 per experimental condition for each experiment).

To explore the therapeutic benefit of using combinations of immunomodulatory tumor-derived cells, we performed *in vivo* experiments using immunocompetent animals challenged with the syngeneic B16 tumor-derived melanoma. The parental B16 cells were given subcutaneously, followed by irradiated immunomodulatory B16F10-derived cells (Figure 5A). Our results suggest that all combinations were able to boost an antitumor immune response. When we analyze the survival fractions (Figure 5B), it is possible to identify three groups of the antitumor response. The group without treatment and the lowest survival fraction that was only given 1× PBS, the group treated with the triple combination (4-1BBL + OX40L + GM-CSF) that had an intermediate response and, finally, a group with the highest survival fraction, that was given the double combinations (4-1BBL + OX40L, GM-CSF + 4-1BBL, GM-CSF + OX40L).

In addition, it was observed that all the combinations showed an elevated ratio of CD8/Treg in tumor sites when compared to non-treated animals (Figure 5C; Figure S2 in Supplementary Material). An examination of tumor histology has shown an increased infiltration of CD8 T cells in treated animals when compared to non-treated animals (Figures 5D,E).

### Combining Immunomodulatory Tumor-Derived Vaccines Induces an Increased Protective Effect in Cured and Rechallenged Animals

**Figure 6:**
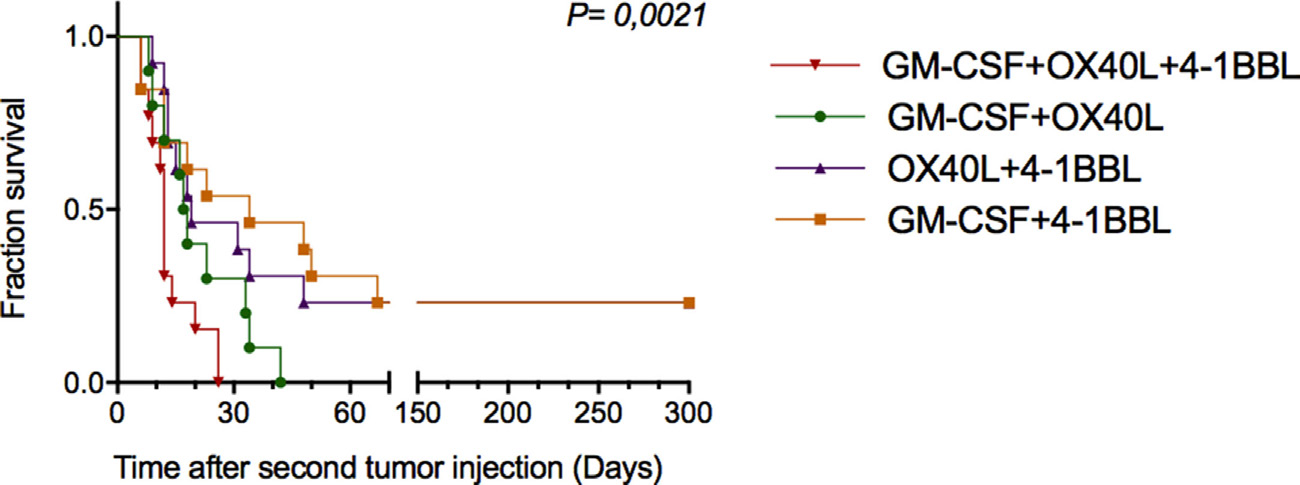
Combinations of immunomodulatory tumor-derived vaccines prevent tumor progression in previously cured and subsequently rechallenged animals. Thirty days after the first challenge, animals that were free of tumors were rechallenged with parental B16F10 cells. The graph shows the cumulative survival curves for three independent experiments (*n* = 12 mice per group). The survival curves were analyzed using the Log-rank test.

To investigate whether antitumor vaccines provide long-term protection, we rechallenged 16 cured animals that were previously challenged once with the combinations. In this way, on day 30 (Figure 5A), these animals were given new injections of parental B16-F10 tumor cells monitoring tumor growth. As shown in Figure 6, we observed increased survival in the animals that received a combination that included cells harboring OX40L plus 4-1BBL and GM-CSF plus 4-1BBL.

## Discussion

Despite therapeutic advances in monoclonal antibody therapies, cases of toxicity and refractory patients continue to be described (11–13). It is therefore necessary to pursue new approaches to improving immunotherapy. In this study, we have explored the effectiveness of combinations of genetically modified tumor cells that express T cell costimulatory ligands and the cytokine GM-CSF. Other groups have described the use of tumor-derived cell lines that coexpress different immunomodulators within the same cell line or the coadministration of tumor vaccines and antibodies (36–39). In contrast, we established different tumor-derived immunomodulatory cell lines, each harboring only a single immunomodulator. We combined these tumor-derived cell lines to investigate synergies to enhance antitumor response. Establishing immunomodulatory cell lines harboring a single immunomodulator increases flexibility to test different combinations. In addition, it is also possible to test combinations using different amounts of immunomodulatory tumor-derived cells and different vaccination protocols for the further investigation of increased therapeutic benefits.

We developed a high-content *in vitro* imaging assay that allowed us to investigate T cell-mediated antitumor responses. This assay is based on counting target cells that simultaneously coexpress an immunomodulator and the GFP reporter gene, in contrast to fluorolysometric assay described in the literature in which target cells encode only the reporter gene (31). In this manner, the same target cell that costimulates T cells can be used as a reporter to monitor T cell-mediated cytotoxicity. Compared to the fluorolysometric assay, the high-content imaging system just uses GFP to count cells and does not depend on measuring fluorescence intensity. This feature is an advantage since GFP is highly stable and can accumulate in the cytoplasm, biasing reproducibility of the assay.

When we performed *in vitro* assays using single immunomodulators, we observed an increased cytotoxic activity mediated by CD8 T cells in tumor-derived cells harboring 4-1BBL, after 96 h (Figure 1). This observation was supported by data in the literature that have associated enhanced T cell activity with the costimulation of 4-1BB (4, 5, 8). However, the cytotoxic effect was enhanced in the presence of combinations of GM-CSF + OX40L, GM-CSF + 4-1BBL, 4-1BBL + OX40L, 4-1BBL + OX40L + GM-CSF (Figure 2). Of note was the fact that some of the immunomodulatory cell lines, or their combinations, were associated with an enhancement of cytotoxicity mediated by preactivated CD4 T cells (Figure 2).

We performed an IFN-γ test in the supernatant of cell cultures (Figure 3) that can suggest a driving of the CD4-T cells to a Th1 phenotype. We observed an increased level of INF-γ for all the combinations of immunomodulatory cell lines and a mild increase in the cytotoxicity induced by single immunomodulatory tumor-derived cells expressing 4-1BBL or GM-CSF (Figure 1, 96 h). The effect of GM-CSF increasing expression of IFN-γ by T cells was already reported in the literature (40). On the other hand, we observed an enhanced cytotoxic effect mediated by CD4 T cells for the double combination GM-CSF + 4-1BBL (Figure 2, 96 h) and the triple combination (Figure 2, 24–96 h). In both of these conditions, we had the highest CD4 mediated cytotoxic effect and detected the highest levels of secreted INF-γ (Figure 3).

The CD4-mediated cytotoxicity is sustained by literature data, suggesting tumor-specific CD4 T cells contribute to B16F10 elimination (41–44). It was also observed that 41BB-L and OX40-L could trigger CD4 T cell cytotoxic response in viral infections (45). In this way, as seen in Figure 2, the triple combination of OX40L, 4-1BBL, and GM-CSF boosts the activity of cytotoxic CD4 T-cells against tumor cells *in vitro*.

It is interesting to note, that animals challenged with monotherapies, using our vaccination protocol, developed tumors faster (data not shown) and the antitumor effect was improved with the combinations, which correlates with the results of the high-content imaging assays. The combinations of antitumor vaccines that encode 4-1BBL and OX40L confer a therapeutic benefit comparable to that of combinations including GM-CSF and 4-1BBL, resulting in the inhibition of tumor growth (Figure 5B). The analysis of T cell infiltrates in the tumor sites revealed that all combinations of tumor-derived vaccines induced an increased CD8 T cell infiltration (Figure 5D) and enhanced the CD8/Treg ratio (Figure 5C). Since Tregs antagonize antitumor response, the inhibition of these cells should contribute to reinforcing antitumor response (46–49).

Although the combination of the three immunomodulatory vaccines has shown a substantial enhancement of T cell mediated cytotoxicity *in vitro*, we did not observe an outstanding effect *in vivo*, when compared with the double combinations. We hypothesized that this observation might be explained by a dose-dependent decrease of therapeutic effect *in vivo*, which is associated with a reduction in the dose of immunomodulatory cells, as supported by the literature (50). Since we used a total amount of one million immunomodulatory cells for all experimental groups, even for single vaccines or combinations, animals vaccinated with the triple combination were given only one-third of each immunomodulatory cells, which could reduce the therapeutic effect. Literature data also report utilization of one million irradiated cells or above, for *in vivo* experiments (41, 51). Some clinical trials, using GVAX also reported a dose-dependent effect that correlates an enhanced therapeutic benefit using a high dose of injected GM-CSF expressing cells, in patients with melanoma (52), prostate cancer (53), non-small-cell lung carcinoma (54) and other tumors (55). Therefore, further experiments are necessary to explore the therapeutic benefit of tumor-derived vaccines employed as triple combinations, increasing the number of injected cells.

A relevant point of this work was to observe the therapeutic benefit of tumor-derived vaccines on re-challenged animals. We observed that a combination of tumor-derived cells harboring 4-1BBL and GM-CSF or 4-1BBL and OX40L conferred protection to rechallenged animals.

These results of tumor inhibition experiments suggest that antitumor vaccination provides an increased protective effect that might enhance immune memory (6). Taken together, these results may contribute to the development of new therapeutic approaches for the treatment of human cancer based on combinations of tumor-derived vaccines harboring immunomodulators.

## Ethics Statement

This study was carried out in accordance to the Laboratory Animal Care regulations. The protocol was approved by the Animal Care and Use Committee (CEUA) from CNPEM, protocol # 15/2015.

## Author Contributions

MB: formulated the original problem, designed experiments, analyzed data, wrote the manuscript, and gave the final approval of the version to be published. AM-R: designed and performed experiments, analyzed data, and wrote the manuscript. CB: assisted *in vitro* experiments. JT: assisted *in vitro* and *in vivo* experiments.

## Conflict of Interest Statement

The authors declare that the research was conducted in the absence of any commercial or financial relationships that could be construed as a potential conflict of interest.
