# Supplementary material for: Exploring Synergy in Combinations of Tumor-Derived Vaccines That Harbor 4-1BBL, OX40L, and GM-CSF
Source: Front Immunol. 2017 Sep 19;8:1150. doi: 10.3389/fimmu.2017.01150 (PMC5610681; doi:10.3389/fimmu.2017.01150)
Supplement: Supplementary file 1 [file image_1.pdf]

Supplementary Material

Exploring synergy in combinations of tumor-derived vaccines that harbor 4-1BBL, OX40L, and GM-CSF

Andrea J. Manrique-Rincón, Camila M. Beraldo, Jéssica M. Toscaro, Marcio C. Bajgelman*

***Correspondence:**
Marcio C. Bajgelman
marcio.bajgelman@lnbio.cnpem.br

FIGURE S1

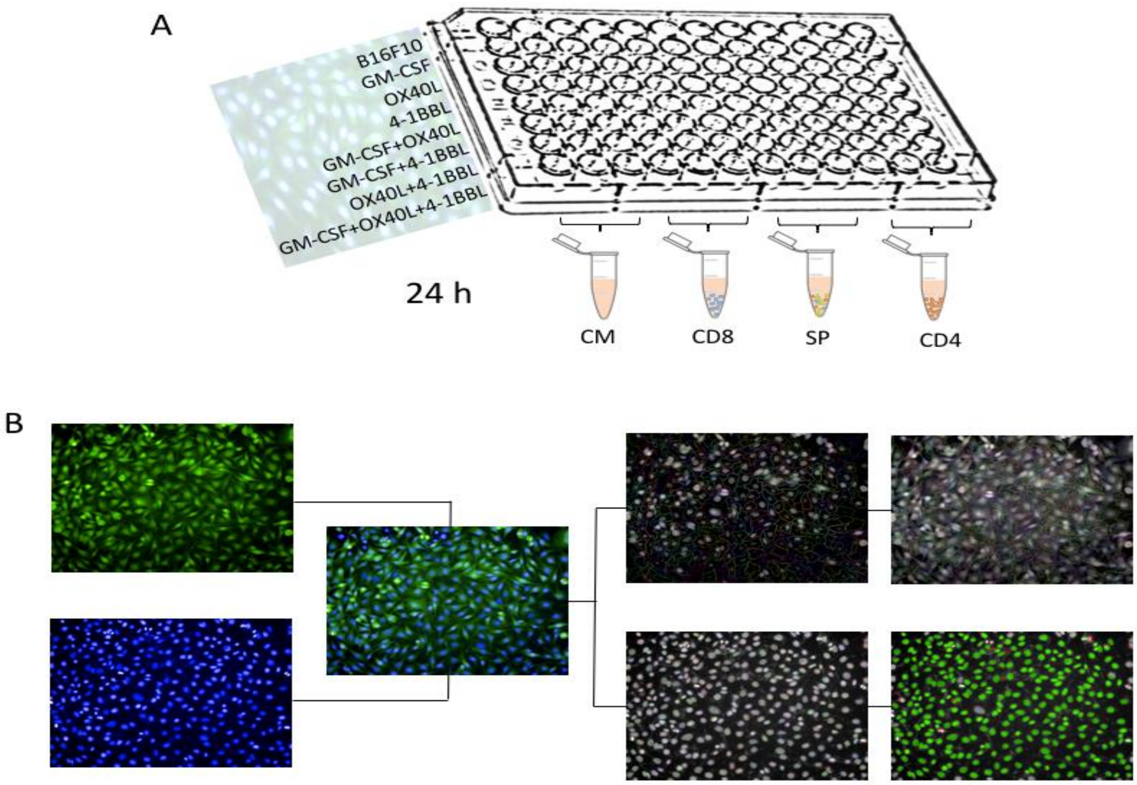

Figure S1. High-content *in vitro* imaging assay used to evaluate effects of tumor cells expressing immunomodulators. Schematic representation of *in vitro* assay (A) tumor modified cells were plated in D10 medium. Twenty-four hours later, when cells where adherent, the D10 medium of tumor cells were changed for complete medium, containing pre-activated CD8, CD4 or freshly isolated splenocytes. Then they were co-cultivated and analyzed using high-content imaging. Analysis of high-content imaging using Operetta system (B). First each well is divided in 27 fields, then each field is acquired for GFP and DAPI, this generates an image that is analyzed based on parameters like roundness for nucleus and fluorescence intensity for GFP to determine the number of live cells in each well.

FIGURE S2

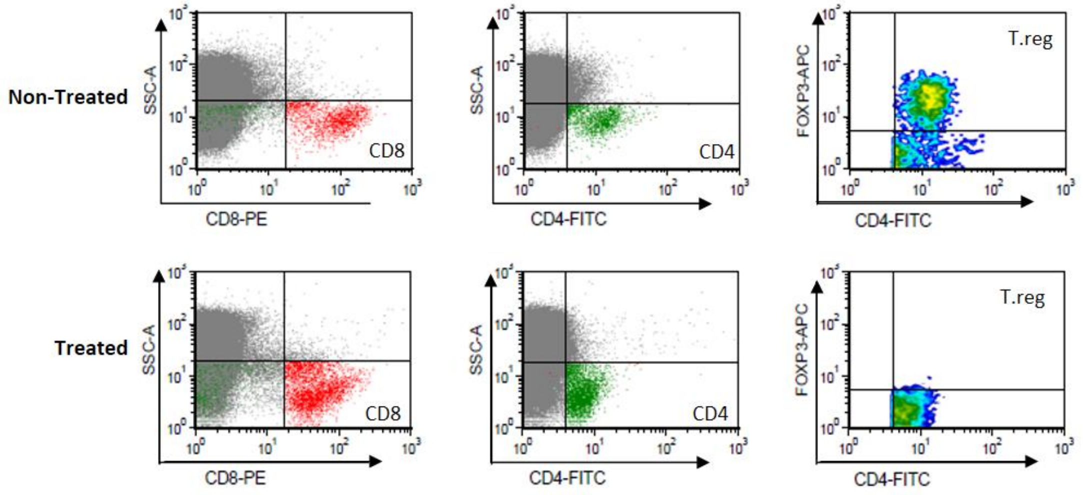

Figure S2. Flow cytometry of tumor infiltrating lymphocytes. Representative dot plots of tumor-infiltrating lymphocytes in non-treated mice (1X PBS) and mice treated with vaccines ((This image is representative of all conditions challenged with double and triple combinations since no significative differences was observed among these groups)). Non treated mice had a small ratio of CD8 (+)Teff /Treg compared to treated mice (Figure 5C). Single-cell suspensions were prepared from tumors at day 28 and stained for CD8, CD4 then cells were fixed, permeabilized and stained for FOXP3.

.
